# Supplementary material for: Identification of photoperiod-induced specific miRNAs in the adrenal glands of Sunite sheep (Ovis aries)
Source: Front Vet Sci. 2022 Jul 22;9:888207. doi: 10.3389/fvets.2022.888207 (PMC9354845; doi:10.3389/fvets.2022.888207)
Supplement: Supplementary file 1 [file Data_Sheet_1.ZIP › Supplementary file/miRDeep2 file.zip!/miRDeep2 file/Know_Structure/SP42A3/oar-mir-329a.pdf]

miRBase precursor : oar-mir-329a
Total read count : 35
oar-miR-329a-5p read count : 7
oar-miR-329a-3p read count : 28
remaining reads : 0

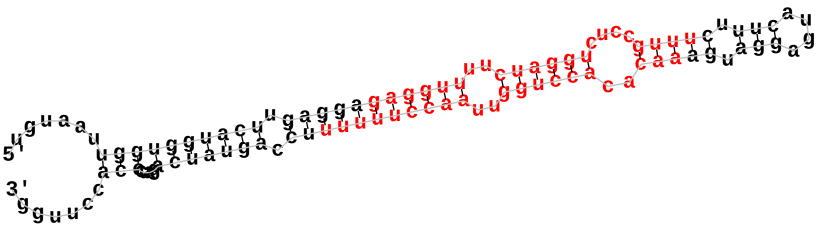

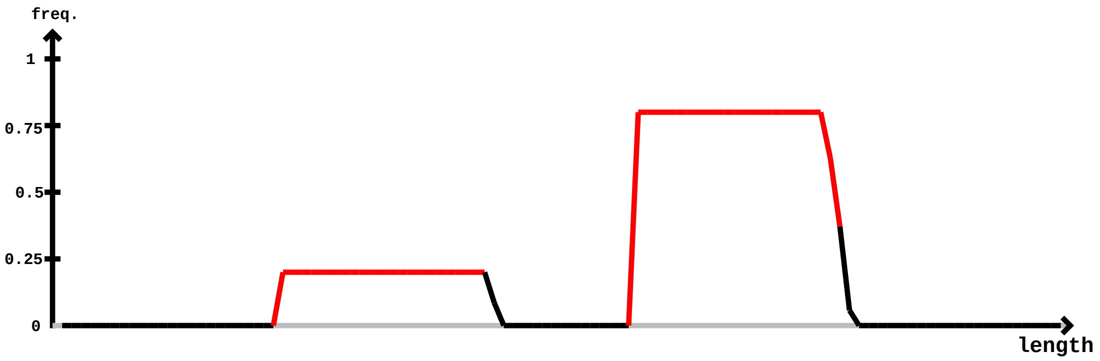


```
                                          oar-miR-329a-5p
                                                                oar-miR-329a-3p
5'- uguaauuggugguacuugaggagagguuuucuaggucuccguuucuuucaugaggaugaaacacaccugguuaaccuuuuuuccaguaucagaucccaccuugg   -3'    exp
    .......(((((((((.(((((((((((..((((((....(((((.(((....))).)))))..))))))..))))))))))).)))).......)))))....   reads      mm         sample
    ......................gagguuuucuaggucuccguuu............................................................   4          0          seq
    ......................gagguuuucuaggucuccguuuc...........................................................   3          0          seq
    ...........................................................aacacaccugguuaaccuuu.........................   5          0          seq
    ...........................................................Cacacaccugguuaaccuuu.........................   1          1          seq
    ...........................................................aacacaccugguuaaccuuuu........................   9          0          seq
    ...........................................................aacacaccugguuaaccuuuuu.......................   11         0          seq
    ...........................................................aacacaccugguuaaccuuuuuu......................   2          0          seq
```
